# Supplementary figures and images for: Important roles of the human leukocyte antigen class I and II molecules and their associated genes in the autoimmune coagulation factor XIII deficiency via whole-exome sequencing analysis
Source: PLoS One. 2021 Sep 10;16(9):e0257322. doi: 10.1371/journal.pone.0257322 (PMC8432773; doi:10.1371/journal.pone.0257322)

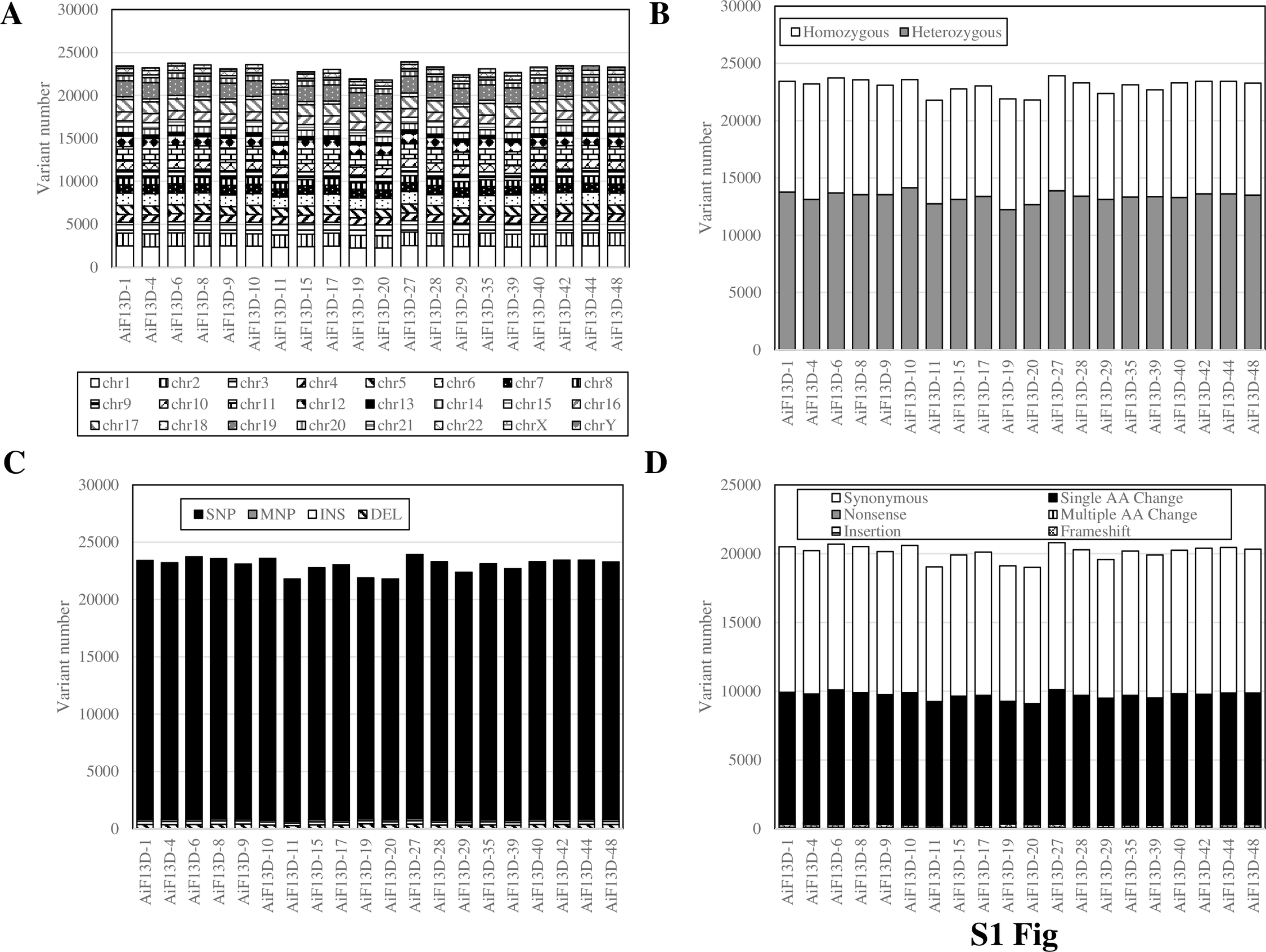

Supplement: S1 Fig — A, Number of variants per chromosomes in each case. B, Number of heterozygous and homozygous variants in each case. C, Number of each genetic variant type in each case. D, Number of each codon mutation type in each case. (TIF) [file pone.0257322.s001.tif]

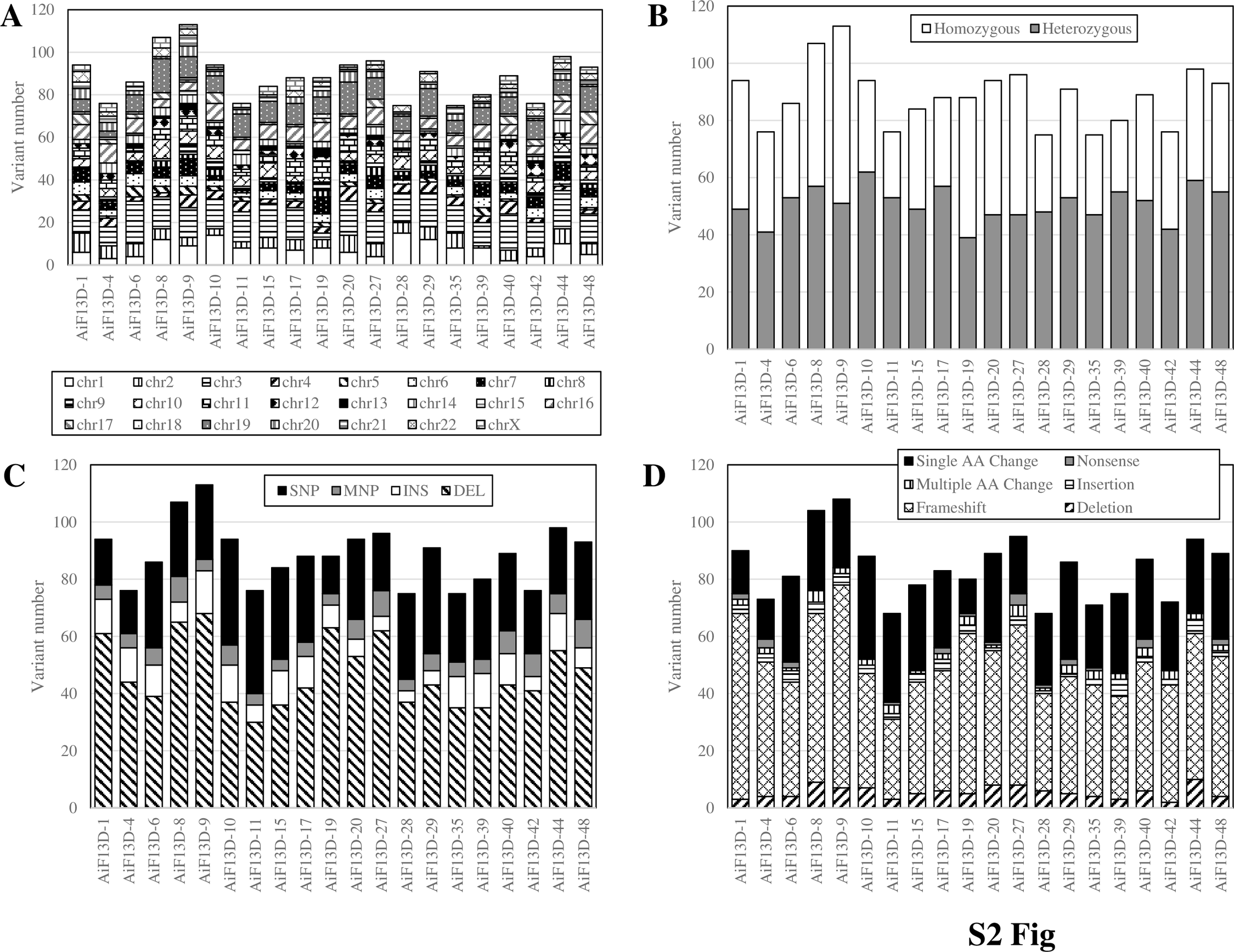

Supplement: S2 Fig — A, Number of variants per chromosomes in each case. B, Number of heterozygous and homozygous variants in each case. C, Number of each genetic variant type in each case. D, Number of each codon mutation type in each case. (TIF) [file pone.0257322.s002.tif]
