# Supplementary material for: Important roles of the human leukocyte antigen class I and II molecules and their associated genes in the autoimmune coagulation factor XIII deficiency via whole-exome sequencing analysis
Source: PLoS One. 2021 Sep 10;16(9):e0257322. doi: 10.1371/journal.pone.0257322 (PMC8432773; doi:10.1371/journal.pone.0257322)
Supplement: Attachment — Submitted filename: Response to Reviewers.docx [file pone.0257322.s013.docx]

August 27, 2021

Dear Dr. Arijit Biswas:

We appreciate reviewer’s comments very much, for they help us to improve the quality of our paper considerably. According to the reviewer’s comments, the manuscript has been revised with yellow backgrounds and red letters.

Before responding point-by-point, we would like to draw your attention to two major additional changes that were revised according to the reviewers’ point out. First, we changed the abbreviations to comply with the nomenclature provided by the Scientific and Standardization Committee of the ISTH. Second, we accept the Reviewer # 2's criticism concerning statistically underpowered analysis, and significantly rewritten “Association of selected candidate alleles with FXIII inhibitory titers and/or levels of anti-FXIII-A autoantibodies” in the “Results” section.

Response to Reviewer #2

Comment 1–1: The nomenclature used throughout the paper is still unacceptable and if the authors are not willing to comply with the nomenclature provided by the Scientific and Standardization Committee of the ISTH (Kohler et al, JTH 2011;9:1404–6), I strongly believe that they should not submit and try to publish in Plos One.

Reply to Comment 1–1: Following the comments of Reviewer #2, we changed the abbreviations to comply with the nomenclature provided by the Scientific and Standardization Committee of the ISTH (Kohler et al, JTH 2011;9:1404–6). Specifically, FXIII/13 was changed to FXIII (94 places including p. 2, lane 3). In addition, AiF13D and AiF8D were changed to autoimmune FXIII deficiency (74 places including p. 2, lane 2) and AHA (9 places including p.7, lane 15).

Comment 1–2: I think that this a major point as by not using the correct nomenclature, confusion is generated within the scientific community and the paper will not advance this field.

Reply to Comment 1–2: Reviewer # 2's comments are justified. So, to avoid confusion in the scientific community, we changed the abbreviations according to the correct nomenclature as described in “Reply to Comment 1–1”.

Comment 2: Moreover, my other points (e.g. omission of statistically underpowered analysis) were not taken fully into consideration, and only minor changes (e.g. adding a limitation section) were introduced in the paper.

Reply to Comment 2: We accept the Reviewer # 2's criticism concerning statistically underpowered analysis, and have deleted the P-value in Tables 3–5, S9 Table, and Fig. 2. In addition, we made the expression a little less than the assertive tone in “Abstract” (p. 2, lanes 12–17), “Results” (p. 23, lane 15–p. 24, lane 9) and “Discussion” (p. 28, lanes 5–11). The relevant parts of the rest of the manuscript have been altered accordingly (p. 14, lane 1 & legends of Tables 3–5 and S9 Table).

Comment 3: I believe that the paper in its current form is misleading both due to the nomenclature used but also due to contents that are not supported by sound statistical analysis and thus, unfortunately, I cannot agree to the publication of this manuscript in its current form.

Reply to Comment 3: Following the comments of Reviewer #2, we changed the abbreviations to comply with the nomenclature provided by the Scientific and Standardization Committee of the ISTH. In addition, as described in “Reply to Comment 2”, we accept the Reviewer # 2's criticism concerning statistically underpowered analysis, and have deleted the P-value in Tables 3–5, S9 Table, and Fig. 2 to avoid misleading. We believe that the revised manuscript is now suitable for publication.

Response to Reviewer #3

Comment 1: Please could the authors provide the information about the antigen epitope of anti-FXIII/13 antibody (from Prof. Reed's gift).

Reply to Comment 1: Unfortunately, we do not have information about the antigen epitope of the anti-FXIII antibody. However, since this antibody recognizes FXIIIa and FXIII-A_2_B_2_, it is considered that it does not recognize the activation peptide or the binding site with FXIII-B.

Comment 2: For the quantification of FXIII/13 inhibitory titer, the healthy individual controls should be included.

Reply to Comment 2: I would like to include data for healthy individual controls in order to increase the number, but the inhibitory titer of healthy individual controls cannot be calculated. One Bethesda unit is defined as the amount of inhibitor in 1 mL of plasma that will neutralize 50% of the FXIII activity (residual activity = 50%). The numbers of Bethesda units are calculated according to the dilution of the patient's plasma. To determine the BU/ mL, it is necessary to obtain the residual activity between 25 and 75%. However, FXIII activity of healthy individual controls is generally >70%, and the FXIII activity of 1:1 mixture of standard plasma and healthy individual control plasma is >75%. Therefore, we cannot calculate FXIII inhibitory titer of healthy individual controls.

Comment 3: Is it only limited in FXIII/13 A chain for the detection of against FXIII/13 autoantibodies. Please could the authors comment or illustrate on why autoantibody against FXIII/13 is not involved in FXIII/13 B chains.

Reply to Comment 3: Ninety-five percent of the cases with the autoimmune FXIII deficiency we have identified were due to autoantibodies against FXIII-A and the remaining 5% were due to autoantibodies against FXIII-B. As described in S1 Table, case No. 48 used in this study was due to autoantibodies against FXIII-B. However, in these cases, the decrease in the level of FXIII antigen due to clearance is the cause of the decrease in activity, and since there is no neutralizing activity, there is almost no inhibitory titer.

Comment 4: Please could the authors supplement the diagnostic criteria on acquired autoimmune FXIII/13 deficiency or reference in your study series.

Reply to Comment 4: All patients with acquired autoimmune FXIII deficiency met the diagnostic criteria from the ISTH/SSC FXIII/Fibrinogen Subcommittee (Ichinose A et al, Thromb Haemost 2016; 116: 772–774). Following the comments of Reviewer #3, we supplemented the reference in the “Clinical samples” section of the “Materials and methods” section (p. 7, lane 7).

Comment 5: In your present studies, you used two methods (ELISA & IST) for evaluating FXIII/13 autoantibodies levels. Could you please determine which method is more sensitive and specific in identifying autoantibodies against FXIII/13; How about the correlation of the both methods.

Reply to Comment 5: As mentioned in the “Discussion” section (p. 31, lane 5–p. 32, lane 2), we believe that the ELISA method is more specific in identifying autoantibodies against FXIII-A. In fact, the logarithm of the FXIII inhibitory titer was better correlated with the ELISA method (R=0.86, P<0.0001) than with the ICT method (R=0.63, P=0.0037). However, for patients with many low-affinity antibodies to FXIII-A, the ICT method may be more sensitive. The correlation between the two methods was moderately good (R=0.61, P=0.0043).
